# Grip strength cut-points from the Swiss DO-HEALTH population

**DOI:** 10.1186/s11556-023-00323-6

**Published:** 2023-08-05

**Authors:** Michael Gagesch, Maud Wieczorek, Lauren A. Abderhalden, Wei Lang, Gregor Freystaetter, Gabriele Armbrecht, Reto W. Kressig, Bruno Vellas, René Rizzoli, Michael Blauth, E. John Orav, Andreas Egli, Heike A. Bischoff-Ferrari

**Affiliations:** 1 Department of Aging Medicine and Aging Research, University Hospital Zurich and University of Zurich, Zurich, Switzerland; 2 Center On Aging and Mobility, University Hospital Zurich, City Hospital Zurich Waid and University of Zurich, Zurich, Switzerland; 3 Department of Radiology, Charité-Universitätsmedizin Berlin, Corporate Member of Freie Universität Berlin and Humboldt-Universität Zu Berlin, Berlin, Germany; 4 University Department of Geriatric Medicine FELIX PLATTER, Basel, Switzerland; 5 UMR INSERM 1027, Gérontopôle, Toulouse University Hospital, University of Toulouse, Toulouse, France; 6 IHU HealthAge, University Hospital Toulouse, Toulouse, France; 7 Service of Bone Diseases, Geneva University Hospitals and Faculty of Medicine, Geneva, Switzerland; 8 Medical University of Innsbruck, Innsbruck, Austria; 9 Department of Medicine, Harvard Medical School, Boston, MA USA; 10 University Clinic for Acute Geriatric Care, City Hospital Waid and Triemli, Zurich, Switzerland

**Keywords:** Physical Performance, Muscle, Sarcopenia, Frailty, Older Adults

## Abstract

**Background:**

While grip strength (GS) is commonly assessed using a Dynamometer, the Martin Vigorimeter was proposed as an alternative method especially in older adults. However, its reference values for Swiss older adults are missing. We therefore aimed to derive sex- and age-specific GS cut-points for the dominant and non-dominant hand (DH; NDH) using the Martin Vigorimeter. Additionally, we aimed to identify clinically relevant weakness and assess convergent validity with key markers of physical function and sarcopenia in generally healthy Swiss older adults.

**Methods:**

This cross-sectional analysis includes baseline data from Swiss participants enrolled in DO-HEALTH, a 3-year randomized controlled trial in community-dwelling adults age 70 + . For both DH and NDH, 4 different definitions of weakness to derive GS cut-points by sex and age category (≤ 75 vs. > 75 years) were used: i) GS below the median of the 1^st^ quintile, ii) GS below the upper limit of the 1^st^ quintile, iii) GS below 2-standard deviation (SD) of the sex- and age-specific mean in DO-HEALTH Swiss healthy agers (i.e. individuals without major chronic diseases, disabilities, cognitive impairment or mental health issues) and iv) GS below 2.5-SD of the sex- and age-specific mean in DO-HEALTH Swiss healthy agers. To assess the proposed cut-points’ convergent validity, we assessed their association with gait speed, time to complete the 5 Times Sit-To-Stand (5TSTS) test, and present sarcopenia.

**Results:**

In total, 976 participants had available GS at the DH (mean age 75.2, 62% women). According to the 4 weakness definitions, GS cut-points at the DH ranged from 29–42 and 25–39 kPa in younger and older women respectively, and from 51–69 and 31–50 kPa in younger and older men respectively. Overall, weakness prevalence ranged from 2.0% to 19.3%. Definitions of weakness using the median and the upper limit of the 1^st^ GS quintile were most consistently associated with markers of physical performance. Weak participants were more likely to have lower gait speed, longer time to complete the 5TSTS, and sarcopenia, compared to participants without weakness.

**Conclusions:**

In generally healthy Swiss older adults, weakness defined by the median or the upper limit of the 1^st^ GS quintile may serve as reference to identify clinically relevant weakness. Additional research is needed in less healthy populations in order to derive representative population-based cut-points.

**Trial registration:**

ClinicalTrials.gov Identifier: NCT01745263.

**Supplementary Information:**

The online version contains supplementary material available at 10.1186/s11556-023-00323-6.

## Background

In older adults, low physical performance is associated with accelerated aging and increased mortality [1]. One key component to identify low physical performance and its adverse outcomes in this population is reduced muscle strength (i.e. weakness) measured by grip strength (GS) [2, 3]. While weakness is a key clinical feature of sarcopenia, defined as the loss of muscle mass and function with aging [4], it is also a core criterion of the physical frailty phenotype [5–7].

Considered as an established marker for weakness, low GS has been linked to poor mobility outcomes in older adults, [8, 9] acts as a recognized measure assessing weakness in physical frailty, [10] and helps identify probable sarcopenia [4, 11]. Of note, GS measurement depends on age, sex, and ethnicity and is therefore also geographically divergent [12]. Consequently, while GS is a quick and easy measure in clinical practice, its procedure requires standardized measurement methods and appropriate cut-points suitable to the local population under investigation [13, 14].

Several operational definitions for sarcopenia and physical frailty proposed cut-points for the identification of clinically relevant weakness based on GS measurement [15, 16]. As example, some investigators used GS cut-points defined as 2.5 standard deviations (SD) below the age and sex-specific mean, [17] while the revised European working group on sarcopenia in older people consensus (EWGSOP2) recommends a cut-point of -2.0 SDs compared to normative regional references from healthy young adults [4]. Additionally, relevant weakness in physical frailty has been defined as GS values within the lowest 20% of the investigated population according to the landmark study by Fried and colleagues, [10] and adopted by several subsequent studies [18]. More recently, the Sarcopenia Definitions and Outcomes Consortium (SDOC) published GS cut-points for muscle weakness that were associated with the prediction of slow walking speed (< 0.8 m/s) [19]. This heterogeneity in the definition of low GS has been further highlighted in a systematic literature review including 72 studies [18], leading to possible discrepancies in the detection of clinically relevant weakness across studies.

Further, the choice of instrument may affect the comparability of GS measurements. The hydraulic devices called dynamometers, including the Jamar® Dynamometer (JD) [14] are most commonly used in reporting GS as isometric force in kilograms (kg) or pounds (lb.). However, the Martin Vigorimeter (MV), dynamically measuring the force of compression in kilopascal (kPa) gained more and more attention recently since it appears better tolerated in older adults with often painful arthritic deformities of the hands and wrists [20, 21].

Despite the increasing number of studies on GS, comparative data for the JD and MV approach, i.e. measuring kg vs. kPa is not widely available hindering direct comparison between the two instruments. Correlation coefficients between both instruments ranged from *r* = 0.63 to *r* = 0.86 in a single-center study by Neumann et al. in 339 randomized patients (mean age 49 ± 18.4 years) with symptom-free hands (excluding deformities, degenerative or inflammatory functional limitations of the upper extremities), indicating a moderate to high correlation [22]. In addition, to our knowledge no validated equation has been published for a direct comparison of the two instruments or the units specified (kg vs. kPa).

Therefore, our study aimed to examine sex- and age-specific GS cut-points in kPa using the Martin Vigorimeter for the dominant and non-dominant hand (DH; NDH) to identify clinically relevant weakness in generally healthy Swiss older adults, and to assess their convergent validity with additional markers of physical function.

## Methods

### Study design and participants

We performed a cross-sectional analysis of baseline data of Swiss older adults included in DO-HEALTH, a three-year randomized controlled clinical trial in adults age 70 years and older from five European countries (NCT01745263) [23]. Swiss participants were recruited in three study centers: Basel (*n* = 253), Geneva (*n* = 201), and Zurich (*n* = 552). Inclusion criteria were absence of major health events (i.e. cancer or myocardial infarction) in the five years prior to enrollment, sufficient mobility to come to the study centers without help, and good cognitive status (defined by a Mini-Mental State Examination (MMSE) score of at least 24 points). The DO-HEALTH trial protocol has been previously published [23].

The Cantonal Ethics Committee of the Canton of Zurich approved our study (BASEC Nr. 2012–0249).

### Grip strength measurement

GS in DO-HEALTH was measured using a standardized method in accordance with the American Society of Hand Therapists (ASHT) recommendations [14]. We report GS readings in kPa at both, DH and NDH, obtained from the best of three consecutive measurements by a calibrated Martin Vigorimeter (KLS Martin KG, Tuttlingen, Germany). According to the standardized DO-HEALTH protocol, GS was measured by trained and certified study personnel in a seated position with the elbow flexed at 90 degrees, forearm in neutral position, parallel to the floor, and with intervals of 30 s between each trial. If a participant had an acute flare-up of arthritis in the hand, a surgery or any other serious injury in the last 2 months, the grip strength test was not be performed on the affected hand. Adherence to the study protocol was overseen by local study visits of the coordinating center as described elsewhere [23]. In addition, we considered the mean of the two best trials of GS measurement in a sensitivity analysis to assess the robustness of the results.

### Definitions of clinically relevant weakness

GS cut-points to identify clinically relevant weakness by sex and age group (i.e. below and above the mean age of our study population) were derived from the following four different operational definitions. First, described within the frailty phenotype concept by Fried et al. [10]: i) below the median of the 1st quintile of GS, ii) below the upper limit of the 1^st^ quintile of GS. Second, within the concept of the EWGSOP2 consensus [4]: iii) 2.0 SD below the sex- and age-specific mean GS in the DO-HEALTH Swiss healthy agers and iv) 2.5 SD below the sex- and age-specific mean GS in the DO-HEALTH Swiss healthy agers. For the two last approaches, we use age- and sex-specific mean GS values of the Swiss healthy agers included in DO-HEALTH, in the absence of an external reference sample from younger Swiss adults. As per the definition used in the Nurses’ Health Study, participants were classified as health agers if they had no major chronic diseases, no disabilities, no impairment in cognitive function, and no mental health limitations [24].

### Assessment of convergent validity

In order to assess the convergent validity of the proposed GS cut-points, we investigated the association of prevalent weakness with markers of lower extremity function, i.e. gait speed (m/s), low gait speed (defined as walking speed < 0.8 m/s and < 1.0 m/s) [19], time to complete the 5 Times Sit-to-Stand (5TSTS) test (s), and longer time to complete the 5TSTS test (> 11.19 s.) [25–27]. In addition, we investigated the association of weakness with the operational definition of sarcopenia by Baumgartner et al. (appendicular lean mass divided by height squared, ≥ 2 standard deviations below sex-specific means of the Rosetta study) [28], among participants enrolled in Zurich, which was the one Swiss study site performing whole body composition measurement (Lunar iDXA; GE Healthcare). Appendicular lean mass (ALM) calculation was based on the sum of lean mass in both arms and legs. Relative ALM was calculated as ALM divided by body height in meters squared.

### Statistical analysis

The baseline characteristics of our study sample are described overall and by sex. Normally distributed continuous variables are presented as mean and standard deviation (SD) and non-normal variables as median and interquartile range (IQR). Categorical variables are presented in frequencies and percentages. Differences between men and women at baseline were tested using the Wilcoxon rank sum test, t-test, or chi-square test, for non-normal, normal, and categorical variables, respectively.

The reliability of the GS measurement at the DH and NDH was assessed with intra-class correlation coefficients, with values greater than 0.90 indicating excellent reliability [29]. The distribution of GS at the DH and NDH was examined among Swiss healthy agers and all Swiss participants. To account for outliers and potential measurement or data-entry errors, we excluded individuals with GS values lower than the 1st quartile minus 1.5 times the interquartile range or greater than the 3rd quartile plus 1.5 times the interquartile range. Given the normal distribution of GS at the DH and NDH (skewness < 1.5), we used t-tests to compare mean GS by sex and age group.

To assess convergent validity, differences in mean gait speed and time to complete the 5TSTS test between participants with and without weakness were tested using t-tests. Differences in the prevalence of low gait speed, longer time to complete the 5TSTS test, and sarcopenia between participants with and without weakness were tested using Chi-square or Fischer’s exact tests, in case of small sample sizes. Statistical significance was assumed at *P* < 0.05. All statistical analyses were performed using SAS v9.4 (SAS Institute, Inc., Cary, NC, United States).

## Results

### Baseline characteristics of the study population

Of the 1,006 Swiss DO-HEALTH trial participants, three and seven had missing baseline GS measurements at the DH and NDH, respectively. After excluding 27 outliers for GS at the DH and 22 outliers for GS at the NDH due to potential measurement or data-entry errors, data from 976 and 977 participants were used to derive GS cut-points at the DH and NDH, respectively.

The baseline characteristics of the 976 participants with available GS at the DH are presented in Table 1. Overall, the mean age was 75.2 (4.6) years and 61.8% were women. The mean BMI was 25.8 (4.2) kg/m^2^. Participants had good lower extremity function with a median Short Physical Performance Battery score of 12.0 (11.0–12.0) and a mean gait speed of 1.1 (0.2) m/s. The prevalence of sarcopenia was 12.9%.

**Table 1:** Baseline characteristics of the study population

	**Grip strength at the dominant hand**				**Grip strength at the non-dominant hand**			
	**Women**	**Men**	*P* values	**Overall**	**Women**	**Men**	*P* values	**Overall**
	*N* = 603 (61.8%)	*N* = 373 (38.2%)		*N* = 976	*N* = 600 (61.4%)	*N* = 377 (38.6%)		*N* = 977
Age, mean (SD), years	75.2 (4.5)	75.4 (4.8)	0.45	75.2 (4.6)	75.1 (4.5)	75.4 (4.8)	0.35	75.2 (4.6)
Age groups, N (%)								
≤ 75 years	367 (60.9)	221 (59.2)	0.62	588 (60.2)	366 (61.0)	223 (59.1)	0.57	589 (60.3)
> 75 years	236 (39.1)	152 (40.8)		388 (39.8)	234 (39.0)	154 (40.9)		388 (39.7)
Height, mean (SD), m	1.6 (0.1)	1.7 (0.1)	< .001	1.6 (0.1)	1.6 (0.1)	1.7 (0.1)	< .001	1.7 (0.1)
Weight, mean (SD), kg	65.4 (12.0)	78.3 (11.6)	< .0001	70.3 (13.4)	65.5 (11.9)	78.4 (11.8)		70.5 (13.4)
BMI^b^, mean (SD), kg/m^2^	25.5 (4.5)	26.3 (3.6)	0.003	25.8 (4.2)	25.5 (4.5)	26.3 (3.6)	0.002	25.8 (4.2)
SPPB score^c^, median (IQR)	12.0 (11.0–12.0)	12.0 (11.0–12.0)	0.004	12.0 (11.0–12.0)	12.0 (11.0–12.0)	12.0 (11.0–12.0)	0.005	12.0 (11.0–12.0)
Gait speed, mean (SD), m/s	1.1 (0.2)	1.1 (0.2)	0.23	1.1 (0.2)	1.1 (0.2)	1.1 (0.2)	0.16	1.1 (0.2)
5 times sit-to-stand test, mean (SD)	11.0 (3.0)	10.5 (3.0)	0.006	10.8 (3.0)	11.0 (3.0)	10.5 (3.0)	0.004	10.8 (3.0)
Study centers, N (%)								
Basel	146 (24.2)	89 (23.9)	0.57	235 (24.1)	147 (24.5)	94 (24.9)	0.48	241 (24.7)
Geneva	127 (21.1)	69 (18.5)		196 (20.1)	127 (21.2)	68 (18.0)		195 (20.0)
Zurich	330 (54.7)	215 (57.6)		545 (55.8)	326 (54.3)	215 (57.0)		541 (55.4)
Prevalence of impaired function								
Gait speed < 0.8 m/s (n, %)	50 (8.3)	24 (6.5)	0.29	74 (7.6)	49 (8.2)	24 (6.4)	0.30	73 (7.5)
Gait speed < 1.0 m/s (n, %)	180 (29.9)	119 (32.0)	0.49	299 (30.7)	181 (30.2)	119 (31.7)	0.64	300 (30.8)
Slow 5TSTS > 11.19 s	235 (39.6)	126 (33.9)	0.08	361 (37.4)	235 (39.8)	127 (33.8)	0.06	362 (37.4)
Present sarcopenia^d^ (n, %)	25 (7.6)	45 (21.0)	< .001	70 (12.9)	24 (7.4)	46 (21.5)	< .001	70 (13.0)

Medians and IQRs are presented for variables with skewness > 1.5. Percentages are rounded to 1 decimal, which could lead to percentage sums of 100.1% or 99.9%. ^b^ Body mass index (BMI) was calculated as weight in kilograms divided by height in meters squared. ^c^ The Short Physical Performance Battery (SPPB) assesses lower extremity function. Scores range from 0 to 12, in which higher scores are better, ^d^ Baumgartner definition (RSMI), only assessed at Zurich

### Baseline grip strength at the dominant and non-dominant hands

The reliability of the GS measurements was excellent with intra-class correlation coefficients of 0.97 and 0.98 at the DH and NDH, respectively. Comparing GS by sex and age group, younger women had significantly lower mean GS at the DH and NDH compared to men of the same age group (DH: 54.9 [10.6] vs. 79.9 [11.9], NDH: 51.9 [10.8] vs. 79.9 [11.9]; both *P* < 0.001) and older women had significantly lower mean GS at the DH and NDH compared to men of the same age group (DH: 48.9 [9.6] vs. 70.7 [16.1], NDH: 45.9 [9.4] vs. 67.9 [16.6], both *P* < 0.001).

**Fig. 1:**
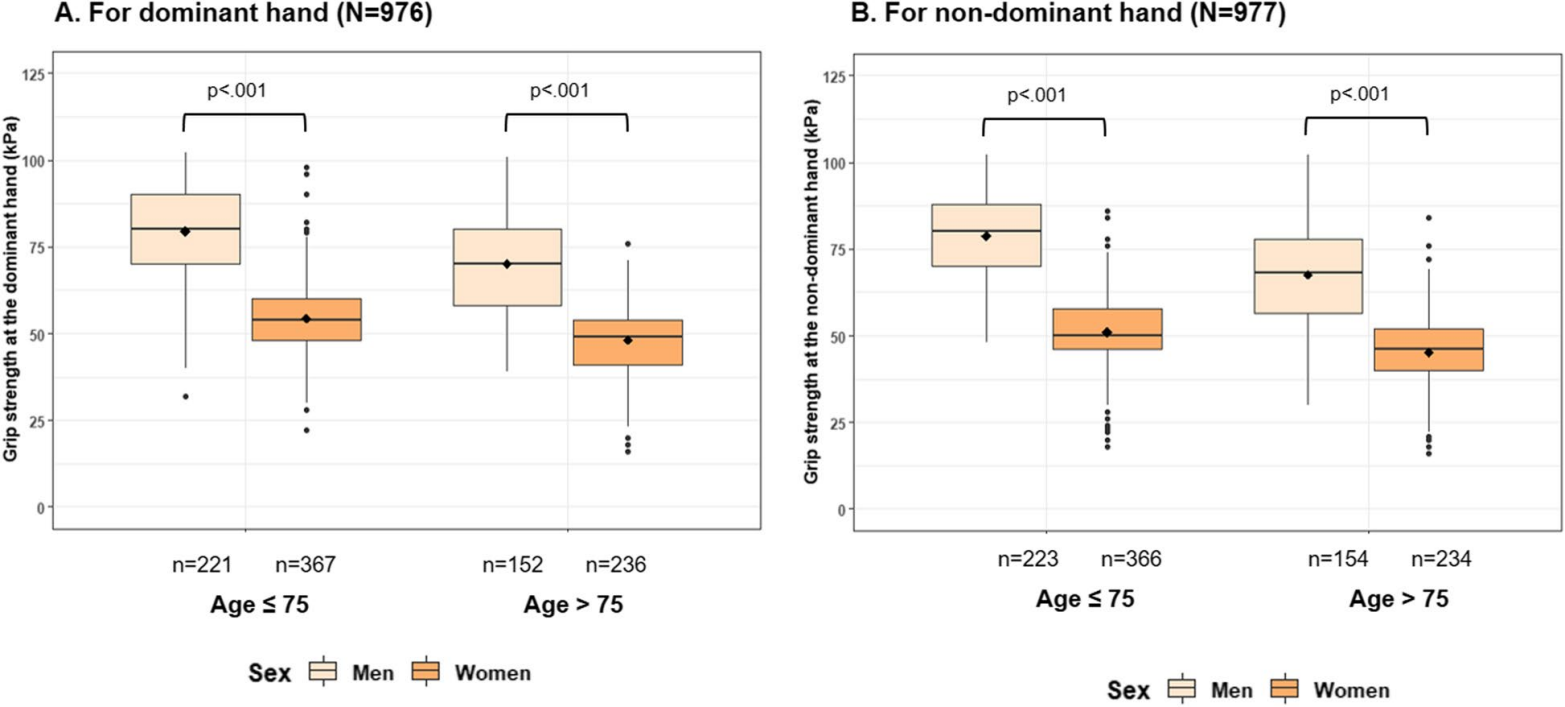
Distribution of grip strength by sex and age group for the dominant hand (**A**) and non-dominant hand (NDH) (**B**)

Figure 1 shows the distribution of GS by sex and age group for DH and NDH. Sex- and age-specific mean GS at the DH and NDH among the Swiss healthy agers are shown in Table 2. The distribution of GS in sex- and age-specific quintiles (Q1-5) for both hands in the study population is further described in Supplementary Table 2a and Supplementary Table 2b. Further, the results of our sensitivity analysis for sex- and age-specific mean GS of the two best trials at the DH and NDH among healthy agers (kPa) were overall consistent with our main analysis, and are presented in Supplementary Table 3.

**Table 2:** Sex- and age-specific mean GS at the dominant and non-dominant hand among healthy agers (kPa)

		*Dominant Hand*		*Non-dominant Hand*	
**Sex**	**Age category**	**Number of healthy agers**	**Mean GS (SD)** **at the DH**	**Number of healthy agers**	**Mean GS (SD)** **at the NDH**
Men	Age ≤ 75	124	80.0 (12.0)	127	79.9 (11.9)
	Age 75 +	63	70.7 (16.1)	63	67.9 (16.6)
Women	Age ≤ 75	218	54.9 (10.6)	218	51.9 (10.8)
	Age 75 +	93	48.9 (9.6)	93	45.9 (9.4)

### Grip strength cut-points and prevalence of weakness

For definition i) (GS below the median of the 1^st^ quintile, GS cut-points for the DH were 42 and 34 kPa for younger and older women respectively, and 64 and 50 kPa for younger and older men. For definition ii) (GS below the upper limit of the 1^st^ quintile), GS cut-points for the DH were 46 and 39 kPa for younger and older women respectively, and 69 and 55 kPa for younger and older men. For definition iii) (below 2-SD of the sex- and age-specific mean GS in the DO-HEALTH Swiss healthy agers), GS cut-points for the DH were 34 and 30 kPa for younger and older women respectively, and 57 and 39 kPa for younger and older men. Finally, for definition iv) (below 2.5-SD of the sex- and age-specific mean GS in the DO-HEALTH Swiss healthy agers), GS cut-points at the DH were 29 and 25 kPa for younger and older women respectively, and 51 and 31 kPa for younger and older men. Our proposed cut-points for clinically relevant weakness according to all four operational definitions are summarized in Table 3.

**Table 3:** Summary cut-points for weakness by sex and age category (kPa) among Swiss participants

		*Dominant hand*		*Non-dominant hand*	
**Proposed cut-point**	**Age category**	**Men**	**Women**	**Men**	**Women**
Below median in lowest 20%	Age ≤ 75	64	42	62	38.5
	Age 75 +	50	34	48.5	30.5
Upper limit of lowest quintile	Age ≤ 75	69	46	67	42
	Age 75 +	55	39	52	37
Below -2 SD of mean	Age ≤ 75	57	34	57	31
	Age 75 +	39	30	35	28
Below -2.5 SD of mean	Age ≤ 75	51	29	51	26
	Age 75 +	31	25	27	23

Overall, the prevalence of weakness was 8.8% and 9.3% at the DH and NDH for definition i), 19.5% and 19.3% at the DH and NDH for definition ii), 3.1% and 2.8% at the DH and NDH for definition iii), and 1.3% and 1.5% at the DH and NDH for definition iv).

### Convergent validity of the proposed cut-points

Our results regarding the convergent validity of the proposed cut-points for weakness by operational definition are summarized in Table 4. When considering GS at the DH, definitions i) and ii) were most consistently associated with the selected markers of physical performance. Compared to participants without weakness, participants with prevalent weakness were more likely to have significantly lower mean gait speed (both *p*-values < 0.001) and to need a longer time to complete the 5TSTS (*p* = 0.001 and *p* < 0.001, respectively). Further, participants with weakness were more likely to have a higher prevalence of low gait speed (all *p*-values < 0.05), of longer time to complete the 5TSTS test (*p* = 0.001 and *p* < 0.001, respectively), and of sarcopenia (*p* = 0.02 and *p* = 0.03, respectively).

**Table 4:** Convergent validity of the proposed cut-points for weakness with markers of physical performance

	**Definition**	**Present weak-ness**	**N**	**Gait speed (m/sec), mean (SD)**	***P* value**	**Low gait speed** ** < 1.0 m/s** **(n, %)**	***P *value**	**Low gait speed** ** < 0.8 m/s** **(n, %)**	***P* value**	**5TSTS (sec), mean (SD)**	***P* value**	**5TSTS** ** > 11.19 s** **(n, %)**	***P* value**	**Present sarcopenia (n, %)**	***P* value**
**Dominant hand**	Below median of lowest 20%	yes	93	1.02 (0.21)	** < .001**	43 (46.24)	** < .001**	14 (15.05)	**0.004**	11.94 (3.43)	**0.001**	47 (51.65)	**0.003**	13 (21.67)	**0.03**
		no	911	1.11 (0.21)		264 (28.98)		61 (6.70)		10.68 (2.92)		325 (35.91)		58 (11.81)	
	Upper limit of lowest quintile	yes	194	1.03 (0.21)	** < .001**	81 (41.75)	** < .001**	29 (14.95)	** < .001**	11.82 (3.57)	** < .001**	92 (49.20)	** < .001**	24 (18.75)	**0.02**
		no	807	1.12 (0.21)		223 (27.63)		46 (5.70)		10.54 (2.78)		269 (34.53)		46 (11.06)	
	Below -2 SD of mean	yes	37	0.99 (0.19)	** < .001**	19 (51.35)	**0.005**	6 (16.22)	0.05	12.29 (3.23)	**0.002**	20 (55.56)	**0.02**	6 (25.00)	0.11
		no	967	1.11 (0.22)		288 (29.78)		69 (7.14)		10.74 (2.97)		352 (36.67)		65 (12.33)	
	Below -2.5 SD of mean	yes	20	0.93 (0.15)	** < .001**	14 (70.00)	** < .001**	3 (15.00)	0.18	12.39 (3.30)	**0.02**	11 (55.00)	0.10	3 (27.27)	0.16
		no	984	1.11 (0.22)		293 (29.78)		72 (7.32)		10.76 (2.98)		361 (36.99)		68 (12.59)	
**Non-dominant hand**	Below median of lowest 20%	yes	102	1.01 (0.22)	** < .001**	44 (43.14)	**0.004**	20 (19.61)	** < .001**	11.58 (2.88)	**0.005**	50 (50.00)	**0.006**	14 (22.95)	**0.01**
		no	902	1.11 (0.21)		263 (29.16)		55 (6.10)		10.70 (2.99)		322 (35.94)		57 (11.63)	
	Upper limit of lowest quintile	yes	192	1.03 (0.23)	** < .001**	82 (42.71)	** < .001**	30 (15.63)	** < .001**	11.54 (3.29)	** < .001**	89 (48.11)	** < .001**	27 (22.31)	** < .001**
		no	805	1.12 (0.21)		223 (27.70)		44 (5.47)		10.62 (2.90)		273 (34.91)		43 (10.26)	
	Below -2 SD of mean	yes	29	0.96 (0.17)	** < .001**	17 (58.62)	** < .001**	5 (17.24)	0.06	12.14 (3.03)	**0.02**	15 (53.57)	0.07	5 (25.00)	0.16
		no	975	1.11 (0.22)		290 (29.74)		70 (7.18)		10.75 (2.98)		357 (36.88)		66 (12.43)	
	Below -2.5 SD of mean	yes	17	0.94 (0.15)	**0.002**	11 (64.71)	**0.002**	2 (11.76)	0.37	12.53 (3.44)	**0.02**	10 (58.82)	0.06	2 (25.00)	0.30
		no	987	1.11 (0.22)		296 (29.99)		73 (7.40)		10.76 (2.98)		362 (36.98)		69 (12.71)	

When using definition iii), participants with weakness were more likely to have a lower mean gait speed (*p* < 0.001), to have a higher prevalence of low gait speed (< 1 m/s, *p* = 0.005), to need a longer time to complete the 5TSTS test (*p* = 0.002 and *p* = 0.02). However, there was no significant difference between participants with and without weakness in the prevalence of sarcopenia (*p* = 0.11). Similar findings were observed for definition iv) and results were fully consistent when considering GS at the NDH.

Results from a sensitivity analysis using the mean of the two best trials of GS measurement to assess the convergent validity of the proposed cut-points for weakness by operational definition were largely consistent with the results from the main analysis and are summarized in Supplementary Table 4.

## Discussion

In our study sample of generally healthy Swiss adults, age 70 years and older who were enrolled in DO-HEALTH, we found significant differences in the distribution of GS with respect to sex and age group (< 75 vs. ≥ 75 years). Based on our analysis, GS cut-points based on the upper limit and the median of the lowest quintile by sex and age group were most consistently associated with markers of physical performance in our sample of generally healthy community-dwelling older adults.

Our approach is comparable to the suggested cut-points identified in the landmark study by Fried et al., where the weakness criterion (stratified by sex and BMI) was fulfilled between 17–21 kg (approximately 32–40 kPa) for women and between 29–32 kg (approximately 51–56 kPa) for men, regardless of age for both sexes [10, 30] Further, the 2018 EWGSOP2 criteria set their sarcopenia cut-off points for low strength in women at 16 kg (approximately 32 kPa) and for men at 27 kg (approximately 50 kPa) of GS [4], and the most recent SDOC cut-points for GS were published with 20 kg (approximately 44 kPa) for women and 35.5 kg (approximately 64 kPa) for men [19, 31]. In this context, it should be acknowledged, that a comparison of current sarcopenia definitions displays major discordances in regard to sarcopenia prevalence [16].

Our results are consistent with these two consensus reports, also with respect to the identification of sarcopenia in the population of generally healthy Swiss older adults enrolled in DO-HEALTH. However, no equation for direct comparison of both instruments used (Jamar Dynamometer vs. Martin Vigorimeter) or the provided units (kg vs. kPa) exists so far, hindering direct comparison.

In regard to the selected markers of physical performance, mean gait speed, gait speed < 1.0 m/s, and mean time for the 5TSTS were significantly associated with present weakness by all four definitions. At the same time, low gait speed < 0.8 m/s, 5TSTS > 11.19 s, and present sarcopenia were only significantly associated with present weakness based on the two definitions build on the lowest 20% approach, highlighting the consistency of this approach with physical performance.

A few limitations need consideration. First, we report findings from voluntary, selected, and generally healthy participants of a randomized clinical trial. Therefore, the grip strength of the DO-HEALTH participants may be higher than the grip strength of individuals included in population-based studies. Second, our detailed breakdown by quintiles, sex, and age groups resulted in relatively low numbers of participants in each subgroup, and therefore results may not be generalizable at large. In this regard, the low prevalence of weakness when using definitions derived from the EWGSOP2 consensus could have affected the conclusions regarding the convergent validity of these cut-offs. Therefore, our findings remain to be confirmed in independent studies. At last, the potential for false positive results may exist since we did not adjust for multiple comparison testing.

However, these limitations are balanced by several strengths. First, we report data from a meticulously executed clinical trial. Second, GS measurements in DO-HEALTH were performed according to a standardized protocol and accomplished at all participating sites by trained and certified study personnel. Third, by investigating GS in a group of generally healthy volunteers age 70 years and over without major health events within the last five years and intact cognitive function, our findings may serve as a reference for a comparable older population from Switzerland.

## Conclusions

In summary, our cross-sectional analysis of baseline data from Swiss DO-HEALTH participants is the first to report in detail on GS measurements in kPa by sex and age group utilizing the Martin Vigorimeter. The present study is the first to suggest cut-points to identify clinically relevant weakness based on four operational definitions using the MV at the DH and NDH in a sample of generally healthy community-dwelling Swiss older adults. Our results contribute valuable insight into the topic in this specific population and are in agreement with previous studies on the association of low GS with other markers of physical function [12]. Furthermore, our operational approach of utilizing the median of the lowest quintile as a pragmatic cut-point for the identification of clinically relevant weakness appeared comparable to the upper limit of the lowest quintile approach and may serve as a reference for simple and easy identification of the weakness component of physical frailty and for the identification of probable sarcopenia in the clinical setting. Additional research in less healthy populations appears needed in order to establish representative population-based cut-points.

### Supplementary Information

**Additional file 1:** **Supplementary Table 1. **Baseline characteristics of Swiss DO-HEALTH participants classified as healthy agers. **Supplementary Table 2a. **Quintiles of grip strength at the dominant hand by sex and age category among Swiss participants. **Supplementary Table 2b. **Quintiles of grip strength at the non-dominant hand by sex and age category among Swiss participants. **Supplementary Table 3.** Sex- and age-specific mean GS of two best trials at the dominant and non-dominant hand among healthy agers (kPa). **Supplementary Table 4. **Convergent validity of the cut-points for weakness derived from the mean GS of the two best trials with markers of physical performance

## Data Availability

In a first step, no data will be made available to researchers external to the DOHEALTH Research Group to allow primary researchers to fully exploit the dataset. The data will be shared in a second step according to a controlled access system.

## Abbreviations

ALM: Appendicular lean mass
BMI: Body mass index
DH: Dominant hand
DXA: Dual-energy X-ray absorptiometry
EWGSOP: European working group on sarcopenia in older people
GS: Grip strength
IQR: Inter quartile range
JD: Jamar Dynamometer
kg: Kilogram
kPA: Kilopascal
MV: Martin Vigorimeter
m/s: Metre per second
NDH: Non-dominant hand
SD: Standard deviation
SDOC: Sarcopenia Definitions and Outcomes Consortium
5TSTS: 5 Times Sit-to-Stand test
Q: Quintile
