# Supplementary material for: Grip strength cut-points from the Swiss DO-HEALTH population
Source: Eur Rev Aging Phys Act. 2023 Aug 5;20:13. doi: 10.1186/s11556-023-00323-6 (PMC10403936; doi:10.1186/s11556-023-00323-6)
Supplement: Supplementary file 1 — Additional file 1: Supplementary Table 1. Baseline characteristics of Swiss DO-HEALTH participants classified as healthy agers. Supplementary Table 2a. Quintiles of grip strength at the dominant hand by sex and age category among Swiss participants. Supplementary Table 2b. Quintiles of grip strength at the non-dominant hand by sex and age category among Swiss participants. Supplementary Table 3. Sex- and age-specific mean GS of two best trials at the dominant and non-dominant hand among healthy agers (kPa). Supplementary Table 4. Convergent validity of the cut-points for weakness derived from the mean GS of the two best trials with markers of physical performance [file 11556_2023_323_MOESM1_ESM.docx]

**Gagesch M, Wieczorek M, Abderhalden LAA, et al; Grip strength cut-points from the Swiss DO-HEALTH population**

**Supplementary File**

**Supplementary Table 1.** Baseline characteristics of Swiss DO-HEALTH participants classified as healthy agers

|  | Swiss healthy agers with available GS at the DH (n=498) | Swiss healthy agers with available GS at the NDH (n=501) |
| --- | --- | --- |
| Women (n, %) | 311 (62.5) | 311 (62.1) |
| Age (mean, SD) | 74.4 (4.2) | 74.4 (4.1) |
|  |  |  |
| Height, m (mean, SD) | 1.7 (0.1) | 1.7 (0.1) |
| Weight, kg (mean, SD) | 68.7 (12.2) | 69.0 (12.3) |
| BMI (kg/m^2^) (mean, SD) | 25.1 (3.7) | 25.2 (3.7) |
| SPPB sum score (median, IQR) | 12.0 (11.0-12.0) | 12.0 (11.0-12.0) |
| Chair rise test, sec. (mean, SD) | 10.2 (2.4) | 10.1 (2.4) |
| Gait speed, m/s (mean, SD) | 1.2 (0.2) | 1.2 (0.2) |
| ***Prevalence of impaired functioning parameters*** |  | |
| Gait speed <0.8m/s (n, %) | 11 (2.2) | 11 (2.2) |
| Gait speed <1.0m/s (n, %) | 104 (21.0) | 103 (20.6) |
| Slow chair rise >11.19s | 144 (29.1 | 142 (28.5) |
| Present sarcopenia (n, %) | 36 (13.4) | 36 (13.3) |
| Outliers for DH: n= 13; Outliers for NDH: n= 10  Outliers were defined at grip strength values below the 1^st^ quartile minus 1.5-times the interquartile range (IQR) or greater than the 3^rd^ quartile plus 1.5-times the IQR; Gait speed missing n=2, chair rise missing n=3, sarcopenia missing n=229 (DH) and n=230 (NDH) | | |

**Supplementary Table 2a.** Quintiles of grip strength at the dominant hand by sex and age category among Swiss participants

| **Quintile** | **Women ≤75 years (n=367)** | | | | | | **Quintile** | **Women >75 years (n=236)** | | | | | |
| --- | --- | --- | --- | --- | --- | --- | --- | --- | --- | --- | --- | --- | --- |
|  | ***n*** | ***Median*** | ***Mean*** | ***SD*** | ***Min*** | ***Max*** |  | ***n*** | ***Median*** | ***Mean*** | ***SD*** | ***Min*** | ***Max*** |
| *Q1* | 75 | 42.0 | 40.7 | 5.1 | 22.0 | 46.0 | *Q1* | 39 | 34.0 | 32.0 | 6.3 | 16.0 | 39.0 |
| *Q2* | 71 | 50.0 | 49.3 | 1.1 | 47.0 | 51.0 | *Q2* | 57 | 42.0 | 42.5 | 2.3 | 40.0 | 46.0 |
| *Q3* | 77 | 54.0 | 53.8 | 1.5 | 52.0 | 56.0 | *Q3* | 52 | 50.0 | 49.2 | 1.0 | 47.0 | 50.0 |
| *Q4* | 70 | 59.5 | 59.1 | 1.2 | 57.0 | 61.0 | *Q4* | 42 | 53.5 | 53.4 | 1.7 | 51.0 | 56.0 |
| *Q5* | 74 | 66.0 | 69.1 | 7.7 | 62.0 | 98.0 | *Q5* | 46 | 62.0 | 63.2 | 4.5 | 57.0 | 76.0 |
| **Quintile** | **Men ≤75 years (n=221)** | | | | | | **Quintile** | **Men >75 years (n=152)** | | | | | |
|  | ***n*** | ***Median*** | ***Mean*** | ***SD*** | ***Min*** | ***Max*** |  | ***n*** | ***Median*** | ***Mean*** | ***SD*** | ***Min*** | ***Max*** |
| *Q1* | 45 | 64.0 | 60.5 | 8.4 | 32.0 | 69.0 | *Q1* | 31 | 50.0 | 49.3 | 4.7 | 39.0 | 55.0 |
| *Q2* | 44 | 72.5 | 72.7 | 2.4 | 70.0 | 77.0 | *Q2* | 33 | 62.0 | 61.9 | 3.2 | 56.0 | 66.0 |
| *Q3* | 43 | 80.0 | 80.1 | 1.3 | 78.0 | 82.0 | *Q3* | 25 | 70.0 | 70.5 | 1.6 | 68.0 | 73.0 |
| *Q4* | 41 | 88.0 | 87.2 | 2.7 | 83.0 | 91.0 | *Q4* | 33 | 78.0 | 77.4 | 2.2 | 74.0 | 80.0 |
| *Q5* | 48 | 96.5 | 96.7 | 3.2 | 92.0 | 102.0 | *Q5* | 30 | 92.0 | 92.4 | 6.7 | 81.0 | 101.0 |

**Supplementary Table 2b.** Quintiles of grip strength at the non-dominant hand by sex and age category among Swiss participants

| **Quintile** | **Women ≤75 years (n=366)** | | | | | | **Quintile** | **Women >75 years (n=234)** | | | | | |
| --- | --- | --- | --- | --- | --- | --- | --- | --- | --- | --- | --- | --- | --- |
|  | ***n*** | ***Median*** | ***Mean*** | ***SD*** | ***Min*** | ***Max*** |  | ***n*** | ***Median*** | ***Mean*** | ***SD*** | ***Min*** | ***Max*** |
| *Q1* | 74 | 38.5 | 36.0 | 6.5 | 18.0 | 42.0 | *Q1* | 44 | 30.5 | 29.5 | 5.8 | 16.0 | 37.0 |
| *Q2* | 68 | 48.0 | 46.6 | 1.7 | 43.0 | 48.0 | *Q2* | 55 | 40.0 | 40.4 | 1.2 | 38.0 | 42.0 |
| *Q3* | 80 | 50.0 | 50.7 | 1.2 | 49.0 | 53.0 | *Q3* | 44 | 46.0 | 46.1 | 1.5 | 43.0 | 48.0 |
| *Q4* | 69 | 56.0 | 55.9 | 1.7 | 54.0 | 59.0 | *Q4* | 44 | 50.0 | 50.7 | 1.2 | 49.0 | 53.0 |
| *Q5* | 75 | 64.0 | 65.7 | 6.0 | 60.0 | 86.0 | *Q5* | 47 | 58.0 | 59.1 | 6.1 | 54.0 | 84.0 |
| **Quintile** | **Men ≤75 years (n=223)** | | | | | | **Quintile** | **Men >75 years (n=154)** | | | | | |
|  | ***n*** | ***Median*** | ***Mean*** | ***SD*** | ***Min*** | ***Max*** |  | ***n*** | ***Median*** | ***Mean*** | ***SD*** | ***Min*** | ***Max*** |
| *Q1* | 41 | 62.0 | 59.9 | 5.9 | 48.0 | 67.0 | *Q1* | 30 | 48.5 | 45.8 | 6.5 | 30.0 | 52.0 |
| *Q2* | 48 | 70.0 | 70.9 | 2.3 | 68.0 | 75.0 | *Q2* | 31 | 58.0 | 58.5 | 2.8 | 53.0 | 63.0 |
| *Q3* | 50 | 80.0 | 80.0 | 1.9 | 76.0 | 82.0 | *Q3* | 34 | 68.0 | 67.5 | 2.3 | 64.0 | 70.0 |
| *Q4* | 46 | 88.0 | 87.4 | 2.3 | 83.0 | 90.0 | *Q4* | 28 | 76.0 | 75.3 | 3.1 | 71.0 | 81.0 |
| *Q5* | 38 | 96.5 | 96.9 | 2.5 | 91.0 | 102.0 | *Q5* | 31 | 90.0 | 90.8 | 6.2 | 82.0 | 102.0 |

**Supplementary Table 3.** Sex- and age-specific mean GS of two best trials at the dominant and non-dominant hand among healthy agers (kPa)

|  |  | *Dominant Hand* | | *Non-dominant Hand* | |
| --- | --- | --- | --- | --- | --- |
| **Sex** | **Age category** | **Number of**  **healthy agers** | **Mean GS of 2 best trials**  **at the DH**  **Mean (SD)** | **Number of**  **healthy agers** | **Mean GS of 2 best trials**  **at the NDH**  **Mean (SD)** |
| Men | Age ≤ 75 | 124 | 78.6 (11.8) | 127 | 78.7 (11.7) |
|  | Age 75+ | 63 | 69.3 (16.2) | 63 | 66.8 (16.2) |
| Women | Age ≤ 75 | 218 | 53.9 (10.4) | 218 | 51.0 (10.6) |
|  | Age 75+ | 93 | 47.9 (9.3) | 93 | 45.1 (9.2) |

**Supplementary Table 4** Convergent validity of the cut-points for weakness derived from the mean GS of the two best trials with markers of physical performance

|  | **Definition** | **Present weak-ness** | **N** | **Gait speed (m/sec), mean (SD)** | ***P* value** | **Low gait speed**  **<1.0 m/s**  **(n, %)** | ***P***  **value** | **Low gait speed**  **<0.8 m/s**  **(n, %)** | ***P* value** | **5TSTS (sec), mean (SD)** | ***P* value** | **5TSTS**  **> 11.19 s**  **(n, %)** | ***P* value** | **Present sarcopenia (n, %)** | ***P* value** |
| --- | --- | --- | --- | --- | --- | --- | --- | --- | --- | --- | --- | --- | --- | --- | --- |
| **Dominant hand** | Below median of lowest 20% | yes | 100 | 1.01 (0.21) | **<.001** | 16 (16.00) | **<.001** | 46 (46.00) | **<.001** | 11.94 (3.55) | **<.001** | 50 (51.02) | **0.003** | 14 (22.22) | **0.02** |
|  |  | no | 904 | 1.11 (0.21) |  | 59 (6.53) |  | 261 (28.87) |  | 10.67 (2.90) |  | 322 (35.86) |  | 57 (11.68) |  |
|  | Upper limit of lowest quintile | yes | 190 | 1.03 (0.22) | **<.001** | 29 (15.26) | **<.001** | 81 (42.63) | **<.001** | 11.78 (3.57) | **<.001** | 90 (48.13) | **<.001** | 25 (19.53) | **0.01** |
|  |  | no | 783 | 1.12 (0.21) |  | 45 (5.75) |  | 217 (27.71) |  | 10.58 (2.79) |  | 271 (34.83) |  | 45 (10.84) |  |
|  | Below -2 SD of mean | yes | 39 | 0.98 (0.18) | **<.001** | 7 (17.95) | **0.01** | 21 (53.85) | **0.001** | 12.17 (3.219 | **0.004** | 21 (55.26) | **0.02** | 6 (24.00) | 0.12 |
|  |  | no | 965 | 1.11 (0.22) |  | 68 (7.05) |  | 286 (29.64) |  | 10.74 (2.97) |  | 351 (36.64) |  | 65 (12.36) |  |
|  | Below -2.5 SD of mean | yes | 21 | 0.92 (0.15) | **<.001** | 4 (19.05) | 0.07 | 15 (71.43) | **<.001** | 12.39 (3.30) | **0.02** | 11 (55.00) | 0.10 | 3 (25.00) | 0.19 |
|  |  | no | 983 | 1.11 (0.21) |  | 71 (7.22) |  | 292 (29.70) |  | 10.76 (2.98) |  | 361 (36.99) |  | 68 (12.62) |  |
| **Non-dominant hand** | Below median of lowest 20% | yes | 104 | 1.00 (0.22) | **<.001** | 21 (20.19) | **<.001** | 45 (43.27) | **0.003** | 11.68 (3.19) | **0.002** | 51 (50.00) | **0.005** | 14 (22.22) | **0.02** |
|  |  | no | 900 | 1.11 (0.21) |  | 54 (6.00) |  | 262 (29.11) |  | 11.68 (3.19) |  | 321 (35.91) |  | 57 (11.68) |  |
|  | Upper limit of lowest quintile | yes | 189 | 1.03 (0.22) | **<.001** | 29 (15.34) | **<.001** | 81 (42.86) | **<.001** | 11.68 (3.31) | **<.001** | 93 (50.00) | **<.001** | 27 (22.13) | **<.001** |
|  |  | no | 784 | 1.12 (0.21) |  | 44 (5.61) |  | 219 (27.93) |  | 10.61 (2.88) |  | 268 (34.40) |  | 43 (10.29) |  |
|  | Below -2 SD of mean | yes | 57 | 1.01 (0.22) | **0.001** | 11 (19.30) | **0.002** | 21 (36.84) | 0.29 | 11.44 (2.76) | 0.10 | 27 (48.21) | 0.08 | 7 (18.92) | 0.73 |
|  |  | no | 947 | 1.11 (0.21) |  | 64 (6.76) |  | 286 (30.20) |  | 10.75 (3.00) |  | 345 (36.70) |  | 64 (12.45) |  |
|  | Below -2.5 SD of mean | yes | 32 | 1.00 (0.19) | **0.005** | 5 (15.63) | 0.08 | 12 (37.50) | 0.39 | 10.87 (2.95) | 0.88 | 10 (32.26) | 0.55 | 3 (15.00) | 0.77 |
|  |  | no | 972 | 1.11 (0.22) |  | 70 (7.20) |  | 295 (30.35) |  | 10.79 (2.99) |  | 362 (37.51) |  | 68 (12.81) |  |
